# Integrated Theory-Based Health and Development Interventions for Young People: A Global Scoping Review

**DOI:** 10.1177/10901981221130734

**Published:** 2022-10-31

**Authors:** Martha J. Decker, Abigail Gutmann-Gonzalez, Melissa Saphir, Ngoc Tram Nguyen, Qi Zhi, Claire D. Brindis

**Affiliations:** 1 University of California, San Francisco, San Francisco, CA, USA; 2 University of Hawaiʻi at Mānoa, Honolulu, HI, USA

**Keywords:** youth, substance use, violence and victimization, sexual health, literature review

## Abstract

**Background:**

Most health and developmental issues affecting young people are interrelated. However, few interventions address multiple behavioral domains simultaneously or are based on theories that encompass a holistic perspective of youth development.

**Aim:**

The purpose of this scoping review was to identify and describe the range of theory-based, multibehavioral health interventions aimed at improving two or more of the following behavioral youth outcomes: (1) sexual and reproductive health; (2) education and employment; (3) violence; and (4) substance use.

**Methods:**

Interventions conducted worldwide and published in English or Spanish between January 2000 and July 2020 were identified using four databases: PubMed, PsycINFO, LILACS, and SciELO.

**Results:**

A total of 11,084 articles were identified, of which 477 were retrieved and assessed for eligibility. Twenty-three articles (evaluating 21 interventions) ultimately met the inclusion criteria. Most interventions were conducted in the United States and addressed two behavioral domains of interest, although seven interventions incorporated three domains, and one incorporated all four. Substance use was the most common domain (16 interventions) but only in the United States/Canada, followed by sexual and reproductive health (14 interventions). All produced significant improvement in at least one outcome or for at least one subgroup of youth. The most common theoretical foundations were positive youth development and social learning theory.

**Conclusion:**

Integrated interventions that are theory based and evidence informed can support positive development and empower youth to make healthy decisions. Further efforts are needed to address structural and policy issues that affect young people’s developmental opportunities and health outcomes.

Adolescent development is influenced by an array of structural and social determinants of health as well as by individual choices (Marmot & Allen, 2014; Sawyer et al., 2012). The risk and protective factors, such as educational access and employment opportunities, that contribute to one health or developmental outcome often are the same factors that affect other outcomes. For example, multiple studies have established a significant association between poverty and adolescent pregnancy and reduced educational achievement (M. R. Decker et al., 2017; Fatusi & Hindin, 2010). In addition, there is a bidirectional relationship between some health and developmental outcomes. For example, adolescents who drop out of school are more likely to become pregnant, while adolescents who are pregnant or parenting are more likely to drop out of school (Kane et al., 2013). Violence and substance use also share many of the same risk factors at the individual, family, and community level, and are both associated with negative health and developmental outcomes (Newcomb & Locke, 2005; Rivara et al., 2019).

Traditionally, most interventions for young people have focused on reducing one risk behavior, such as violence or substance use, or on improving one developmental outcome, such as academic achievement. Many also have been siloed from other interventions, even when these efforts involve the same population or are in response to the same root causes (M. J. Decker et al., 2015). Given that most of the health and developmental issues affecting young people are interconnected, it is important to better understand the underlying theories, designs, and outcomes of interventions that simultaneously address multiple behaviors.

## Value of Integrated Interventions

According to the Lancet commission on adolescent health and well-being, “the most powerful actions for adolescent health and wellbeing are intersectoral, multilevel, and multi-component” (Patton et al., 2016). Integrated interventions that encompass multiple health and developmental domains may better address young people’s risks related to harmful behaviors and provide opportunities that can positively affect multiple outcomes (Catalano et al., 2012). Integrated interventions can also take advantage of multilevel factors in the family, school environment, and community, which may provide young people with greater adaptability and better health and developmental outcomes than single-behavior or single-level interventions (Hale et al., 2014; Lerner et al., 2011).

One systematic review of interventions intended to reduce multiple risks focused on substance use and risky sexual behaviors. Most of the reviewed studies were school based and located in the United States, with mixed levels of quality and effectiveness (Hale et al., 2014). Another systematic review of school-based interventions found promising evidence for the effectiveness of multibehavioral interventions but identified only limited evidence of a synergistic effect from targeting multiple behaviors at the same time (Busch et al., 2013). A systematic review of girl-centered programs in low- and middle-income countries (LMICs) suggested that multicomponent programs may be more effective than single-component interventions in improving health, social, and economic outcomes (Haberland et al., 2018), although a recent review of interventions to prevent child marriage found that multicomponent interventions were less successful than single-component interventions (Malhotra & Elnakib, 2021).

## Holistic Theories of Youth Development

Interventions that aim to address multiple behavioral domains benefit from incorporating appropriate behavioral change theories and frameworks (Brindis et al., 2005). However, many programs for youth have not explicitly incorporated theories or frameworks into their design (Lopez et al., 2013).

Behavioral theories or frameworks that incorporate a multilevel approach, targeting the individual, family, community, and/or structural context in which youth live and interact, include social learning theory and socioecological theory. Social learning theory (also called social cognitive theory) posits that behavior arises out of “reciprocal determinism”—the ongoing interaction between a person, their behavior, and their environment (Bandura, 1977). Socioecological theory recognizes that individuals’ development and health outcomes are shaped by the multiple nested environments and systems in which they live and interact (Bronfenbrenner, 1979). More recent approaches include empowerment theories, which promote individual agency (Perkins & Zimmerman, 1995), and positive youth development (PYD). The PYD framework recognizes the complexities of adolescence and strives to cultivate healthy development through supportive opportunities and experiences in schools, families, and communities (Damon, 2004).

**Impact Statements**

Health and development interventions for young people have traditionally taken siloed approaches to reducing risk, often adopting a deficit model and focusing on a single behavior, while ignoring related issues. This scoping review identified 21 theory-based programs that integrated multiple health and developmental domains. Although the results show such interventions are feasible, the components of the interventions and the outcomes varied. Additional research is needed to ascertain what theories, components, and implementation approaches are most effective for which adolescent populations and settings.

Several of these theories have been used to design interventions for youth, though the majority have focused on only one behavioral domain. The socioecological model has been used with a variety of public health issues, including violence prevention (Uthman et al., 2010), sexually transmitted infections (DiClemente et al., 2005), and substance use (Elkington et al., 2011). Similarly, interventions employing PYD have improved resiliency and self-efficacy in health domains including substance use and sexual behaviors (Gavin et al., 2010; Lerner et al., 2011).

## Purpose of This Scoping Review

Although prior systematic reviews have assessed interventions in one behavioral domain or are based on a particular theoretical framework, none have reviewed interventions addressing multiple domains using explicit theoretical foundations. The purpose of this scoping review is to locate and examine theory-based, multidomain interventions for young people, summarize key findings, and identify research needed to strengthen future interventions to promote the health and well-being of young people.

## Method

We conducted a scoping review of peer-reviewed articles and gray literature evaluating theory-based interventions or programs that targeted outcomes in two or more health and development domains among youth. We followed the Preferred Reporting Items for Systematic Reviews and Meta-Analyses (PRISMA) guidelines for scoping reviews (Tricco et al., 2018).

### Search Strategy

We searched for studies published in English or Spanish between January 2000 and July 2020 using four online databases: PubMed, PsycINFO, LILACS, and SciELO. We used a combination of search terms and Medical Subject Headings (MeSH; Table 1). (See Supplemental Appendix for the full electronic search strategy for PubMed). In addition, we identified pertinent gray literature by screening publications of key multinational agencies, such as the World Bank and United Nations, and the Blueprints for Healthy Youth Development registry of evidence-based programs (Mihalic & Elliott, 2015). Other sources were identified by manually scanning references of identified sources.

**Table 1.** Search Terms for Included SRH, Violence, Education/Employment, and Substance Use Outcomes.

SRH	Violence	Education/employment	Substance use^a^
Pregnancy, reproductive health services, contraception behavior, sexual behavior, health access, childbearing, contraceptive use	Violence, crime, criminal justice system, violence reduction, dating violence, intimate partner or domestic violence	Job training, technical training, school enrollment, youth idleness, unemployment, student dropout, boredom	Substance use, drug use, substance-related disorders, alcohol, marijuana, inhalants, cocaine, snuff, methamphetamine, opioids, crack, illicit drugs, dependence, narcotics, abuse

*Note.* SRH = sexual and reproductive health.

a Excluded studies that focused only on tobacco use.

The inclusion criteria were as follows:

1. The program addressed two or more of the following health and development domains:
  a. Sexual and reproductive health (SRH)
  b. Education and employment
  c. Substance use
  d. Violence, including perpetration or victimization
2. Participants were 10–24 years old, the World Health Organization’s (2014) definition of young people
3. The research design was a randomized controlled trial (RCT) or quasi-experimental study
4. The program assessed behavioral change, not merely changes in knowledge or attitudes
5. A theory or framework was specified

We excluded pilot studies and descriptions of proposed interventions that had not yet been evaluated. Studies that did not mention a theory or stated only a general “theory of change” were also excluded. When the theory was unclear, the study authors were contacted for further details. If the authors did not respond or did not specify a theory or framework, the study was excluded.

### Study Selection, Data Charting, and Synthesis

Article titles and abstracts were screened independently by three researchers. After initial screening, we retrieved the full text of eligible studies. A few programs had more than one article reporting evaluation findings or involved multiple evaluations conducted over the years. In these cases, we included the most relevant article, such as the one that reported outcomes in two or more domains or had the most robust analysis. The one exception was for a program whose outcomes were published in three separate articles (Gusmões et al., 2018; Sanchez et al., 2017, 2018). Data were extracted from the selected articles using a standardized form. If there was a discrepancy, the researchers reviewed the study and came to a consensus noting reasons for inclusion or exclusion.

The quality of the studies was assessed using the Grading of Recommendations Assessment, Development and Evaluation (GRADE) criteria (Guyatt et al., 2011).

## Results

**Figure 1.**
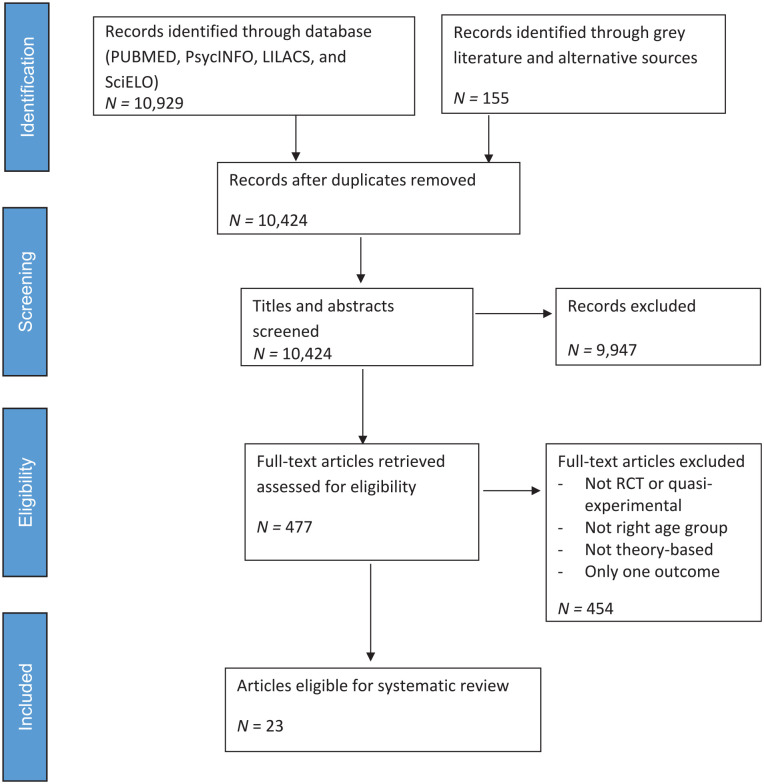
PRISMA flowchart for article identification and screening process. *Note.* PRISMA = Preferred Reporting Items for Systematic Reviews and Meta-Analyses; RCT = randomized controlled trial.

The database and gray literature searches identified 11,084 articles. After removing duplicates, the titles and abstracts of 10,424 articles were screened. Of these, 477 articles were retrieved and assessed for eligibility. A total of 23 articles (reporting on 21 interventions) met the inclusion criteria (Figure 1). All articles meeting the inclusion criteria were published in English.

### Study Characteristics

Table 2 describes the studies that met the inclusion criteria. Seven studies (33.3%) were RCTs with randomization of individuals or families to treatment and control groups. Ten studies (47.6%) were cluster randomized controlled trials (CRCTs), in which classrooms, schools, and/or communities were randomized. Four studies (19.0%) were quasi-experimental without randomization. Study quality ranged from 3 (*moderate*) to 4 (*high*) for RCTs (*M* = 3.7), and from 1 (*very low*) to 4 (*high*) for quasi-experiments (*M* = 2).

**Table 2.** Program Overview by Intervention Type, Country, Theory, and Other Program Components.

Program and study authors	Intervention type	Country	Theory	Participants	Study design	Study quality	Content areas			
							SRH	EE	Sub	Vio
1. Aban Aya Project (Flay et al., 2004)	Two interventions with African American cultural components: (1) 16–21 classroom lessons per year for 4 years; (2) same lessons plus parent, school, and community support	The United States	Triadic influence	*N =* 644 African American youth in Grades 5–8 in 12 urban schools	CRCT	Moderate	✔		✔	✔
2. Adolescent Girls Empowerment Program (Austrian et al., 2020)	(1) Weekly group meetings with mentors teaching SRH, financial education, and life skills for 2 years. (2) Voucher for SRH and general health care. (3) Savings account.	Zambia	Empowerment	*N* = 3,080 girls aged 10–19 years at 5 urban and 5 rural sites	CRCT	High	✔	✔		
3. Better Life Options Program (Centre for Development and Population Activities, 2001)	Six-month vocational skill training, family life education, and individual capacity building through literacy, SRH services, and community involvement	India	Empowerment	*N* = 1,693 women aged 15–26 years	Post-test-only quasi-experiment	Very low	✔	✔		
4. Building Resilience and Vocational Excellence (Griffin et al., 2009)	Substance use and violence prevention classroom curriculum (90-minute lessons 2–3 times per week for 9 weeks), mentoring, peer education, and vocational field trips	The United States	Social learning	*N* = 178 African American youth in 12 Grade 8 classrooms	CRCT	High		✔	✔	✔
5. Children’s Aid Society (CAS)—Carrera model (Philliber et al., 2001)	After-school activities focused on jobs, education, arts, sports, and sexuality knowledge; counseling and discussion groups (5 days a week for up to 3 years)	The United States	PYD	*N* = 1,163 youth aged 13–15 years	RCT	High	✔	✔	✔	✔
6. Communities that Care (Oesterle et al., 2018)	Local stakeholders implemented science-based programs for 5 years that addressed each community’s unique risks	The United States	Social development	*N* = 4,407 youth in Grade 5 in 24 small towns	CRCT	High			✔	✔
7. Familias Unidas (Pantin et al., 2009)	Hispanic-specific, family-based prevention program consisting of nine 2-hour family group sessions and ten 1-hour family visits by trained facilitators	The United States	Social ecological development	*N* = 213 dyads of Hispanic youth in Grade 8 and caregivers	RCT	Moderate	✔		✔	
8. The Fourth R: Skills for Youth Relationships (Wolfe et al., 2009)	21 classroom lessons, information for parents, and student-led committees	Canada	Social cognitive	*N* = 1,722 students in Grade 9 in 20 schools	CRCT	High	✔		✔	✔
9. Girl Empower (Özler et al., 2020)	Weekly lessons on safety, financial literacy, SRH, and other life skills for 39 weeks; savings account; capacity building for local health and psychosocial workers	Liberia	Empowerment	*N* = 1,216 girls aged 13–14 years in 84 communities	CRCT	High	✔	✔		✔
10. Group Psychosocial; Motivational Enhancement Therapy (Bryan et al., 2009)	Three interventions: (1) group psychosocial intervention (GPI) (3 hours), (2) GPI plus motivational enhancement therapy (3–4 hours); (3) information-only control (1 hour)	The United States	Planned behavior	*N* = 484 adolescents in juvenile detention	RCT	High	✔		✔	
11. Health Wise (Smith et al., 2008)	18 classroom lessons over 2 years	South Africa	PYD; ecological systems	*N* = 2,383 youth in Grades 8–9 (*M* age = 14 years)	Quasi-experiment	Low	✔		✔	
12. Healthy Futures (Johnson et al., 2015)	Three 1-hour motivational interviewing sessions focusing on future goals, vocational expectations and planning, career and job exploration, and community resources	The United States	Social cognitive; PYD	*N* = 200 youth aged 14–21 years, primarily African American	RCT	Moderate		✔	✔	✔
13. Iowa Strengthening Families Program (ISFP); Preparing for the Drug Free Years (PDFY) (Spoth et al., 2014)	ISFP: 7-session (13 hours) family-focused intervention teaching communication and problem-solving PDFY: 5-session (10 hours) family competency training	The United States	Risk and protective factors Social development	*N* = 1,309 youth in Grade 6 and their families in 33 schools	CRCT	High	✔		✔	
14. Ishraq (Brady et al., 2007)	Health education, literacy skills, social support, and life skills (3 hours, 4 days per week, up to 30 months); community intervention to create girl-friendly spaces and change gender norms	Egypt	Empowerment	*N* = 587 girls not in school, aged 13–15 years	Quasi-experiment	Very low	✔	✔		
15. Middle School Success (Kim & Leve, 2011)	6 group sessions for caregivers and foster parents and 6 group skill-building sessions for girls; up to 40 individual coaching sessions	The United States	Social learning, social interaction	*N* = 100 girls aged 10–12 years in foster care and their caregivers	RCT	High			✔	✔
16. National Guard Youth Challenge Program (Schwartz et al., 2013)	6-week residential program with classes for GED/diploma, life and job skills training, health components, and community service; mentoring for up to 3 years postprogram	The United States	PYD	*N* = 1,173 youth who dropped out of school, aged 16–18 years	RCT	High		✔	✔	
17. Parents Who Care (Haggerty et al., 2007)	Two trainings for parent and teen: (1) self-administered 108-page workbook and 117-minute video in 18 sections with support by phone; (2) 7 group sessions using the same curriculum and activities (seven 2- to 2.5-hour sessions)	The United States	Social development, social control, social learning, differential association	*N* = 331 Grade 8 African American or European American youth and parents	RCT	Moderate	✔		✔	✔
18. Positive Action (Beets et al., 2009)	35-hour K–12 curriculum, school climate changes, and family and community components annually for 5 years	The United States	Triadic influence	*N* = 1,714 youth in Grade 5 in 20 schools	CRCT	High	✔		✔	✔
19. Project Towards No Drug Abuse (Sussman et al., 2002)	Classroom curriculum for motivation, skills, and decision-making in twelve 40-minute sessions	The United States	Behavioral therapy, social psychology, sociology	*N* = 2,468 youth in Grade 12 in 41 schools	CRCT	High			✔	✔
20. #Tamojunto (Unplugged) (Gusmões et al., 2018; Sanchez et al., 2017, 2018)	Twelve 50-minute lessons covering drug knowledge/attitudes, communication and social skills, and other personal skills	Brazil	Global social influence	*N* = 6,637 youth in Grades 7–8 in 72 schools in 6 cities	CRCT	High			✔	✔
21. Teen Outreach (Allen & Philliber, 2001)	Community service (median hours = 25), class discussions, and class activities on social developmental tasks	The United States	PYD	*N* = 3,277 youth in Grades 9–12	Quasi-experiment	Low	✔	✔		

*Note.* Unless the gender of participants (i.e., “girls” or “boys”) is specified in the Participants column, the studies included girls and boys. SRH = sexual and reproductive health; EE = education and employment; Sub = substance use; Vio = violence; GED = Graduate Equivalency Degree; RCT = randomized controlled trial; CRCT = cluster randomized controlled trial; PYD = positive youth development; ISFP = Iowa Strengthening Families Program.

Sample sizes ranged from 100 to more than 5,000 youth. Only two interventions (9.5%) included participants as young as 10 years, whereas three interventions (14.3%) included youth older than 18 years. Nine programs (42.9%) drew participants from the general population. The others focused on specific racial or ethnic groups (*n* = 5, 23.8%) or on special populations such as girls at risk for poor health or education (*n* = 5, 23.8%), youth who had dropped out of school (*n* = 1, 4.8%), and youth in juvenile detention (*n* = 1, 4.8%). Except for the five studies that focused only on girls, the remaining studies included both boys and girls.

Most interventions (*n* = 14, 66.7%) were conducted in the United States. Seven (33.3%) were conducted in LMICs—Egypt, India, Liberia, South Africa, and Zambia—and the remaining study was conducted in Canada. Six interventions (28.6%) were conducted in urban settings, three (14.3%) in rural settings, and three (14.3%) in both urban and rural settings. Schools were the institutional setting for the majority of the interventions (*n* = 15, 71.4%). Other common settings included community centers (*n* = 4, 19.0%) and participants’ homes (*n* = 3, 14.3%).

The most common theoretical foundations were social learning theory (*n* = 6, 28.6%) and PYD (*n* = 5, 23.8%). Other theories underlying multiple interventions included empowerment theories (*n* = 4, 19.0%), which were used only in LMICs and only among girls; the social development model (*n* = 3, 14.3%); and the theory of triadic influence (*n* = 2, 9.5%).

The most common outcome domain was substance use (*n* = 16, 76.2%), and all but one of the 12 interventions that addressed violence also addressed substance use (*n* = 11, 52.4%). While most studies in the United States/Canada addressed substance use (93%) and violence (60%), only one third of studies in LMICs addressed either of these domains. The second most common domain was SRH (*n* = 14, 66.7%). All the studies (*n* = 21, 100%) reported significant improvement in at least one outcome or one subgroup of youth (Table 3).

**Table 3.** Selected Adolescent Health and Development Outcomes, by Study.

Program	Sexual and reproductive health			Education and employment		Substance use			Violence
	↓Sexual activity	↓Pregnancy	↑Contraception	↑Schooling	↑Employed	↓Alcohol	↓Drugs	↓Combination index	↓Violence
1. Aban Aya	M		M					M	M
2. Adolescent Girls Empowerment	Y	N	N	N					
3. Better Life Options			Y	Y	Y				
4. Building Resilience and Vocational Excellence						M	Y	N	N
5. Children’s Aid Society—Carrera model	N	M	M		Y	N	M		N
6. Communities that Care						M	M		M
7. Familias Unidas	N		Y				Y		
8. The Fourth R: Skills for Youth Relationships			M					N	M
9. Girl Empower	Y	N	Y	N					N
10. Group Psychosocial; Motivational Enhancement Therapy			Y			N			
11. Health Wise	M		N			Y	M		
12. Healthy Futures						N	Y		Y
13. Iowa Strengthening Families; Preparing for the Drug Free Years	Y		Y					Y	
14. Ishraq				Y					
15. Middle School Success								Y	
16. National Guard Youth Challenge				Y	Y	N	N		
17. Parents Who Care	M						M		N
18. Positive Action	Y							Y	Y
19. Towards No Drug Abuse						Y	Y		Y
20. #Tamojunto (Unplugged)						N	M		M
21. Teen Outreach		Y		Y					

*Note.* A blank cell indicates that the outcome was not measured. Y indicates that the main effect for the only measure of concept was significant at *p* < .05, or main effects for all measures of the concept were significant. N indicates that the main effect for the only measure of concept was NOT significant, or main effects for all measures of the concept were not significant. M indicates that the main effect for at least one measure of concept was significant, but main effects for other measures of the concept were not significant, or that there were no main effects, but there was a significant effect in a subpopulation.

The following sections summarize the included interventions and their results according to the number of domains addressed.

### Intervention Addressing Four Domains

The one intervention that addressed all four domains (SRH, violence, education/employment, and substance use) was a multiyear after-school program based on PYD that provided activities focused on support for getting jobs, academic achievement, arts, sports, and SRH education, with the primary goal of reducing teen pregnancy (Philliber et al., 2001). Findings showed that compared with girls in the control arm, significantly more girls in the program used long-acting contraceptives and significantly fewer became pregnant. After the program, participants were more likely to have work experience than controls. While there was a significant reduction in initiating marijuana use among boys, the program did not reduce alcohol use or violence among boys or girls.

### Interventions Addressing Three Domains

#### SRH, Substance Use, Violence

The most common goal of interventions addressing three domains was to improve SRH while reducing substance use and violent behavior (*n* = 4, 19.0%). Only one of these, a 35-hour curriculum based on the theory of triadic influence, with parent involvement and school climate components, produced significant main effects for outcomes in all three domains: reducing sexual activity, substance use, and violent behavior (Beets et al., 2009). The other programs produced effects only in specific subpopulations and/or for some outcomes, but not others. For example, a 16- to 21-lesson classroom curriculum based on the theory of triadic influence and culturally tailored to urban African American youth increased contraception and reduced sexual activity, substance use, and violent behavior, but only among boys who received the curriculum in addition to parent, school, and community components (i.e., not among girls nor among youth who received only the curriculum component; Flay et al., 2004). Similarly, a 21-lesson curriculum based on social cognitive theory in Canada increased contraception and decreased violence among boys (but not among girls), although this program did not affect substance use (Wolfe et al., 2009). A 7-session parent-teen training program based on multiple theories reduced sexual activity and drug use among African American youth, but not among European American youth and had no effect on violence (Haggerty et al., 2007).

#### Education/Employment, Substance Use, Violence

The two programs focused on these domains were shorter than most other reviewed programs, were conducted with relatively small numbers (200 or less) of African American youth, and showed significantly reduced drug use but mixed results for alcohol and violence (Griffin et al., 2009; Johnson et al., 2015). A 9-week program based on social learning theory reduced drug use and one of the two measures of alcohol use, but had no effect on violence (Griffin et al., 2009). In contrast, a 3-hour motivational interviewing intervention based on social cognitive and PYD theories reduced drug use and violent behavior, but had no effect on alcohol use (Johnson et al., 2015). Although both interventions had education/employment components, neither study reported associated outcomes for this domain.

#### SRH, Education/Employment, Violence

One program focused on SRH, education, and violence (Özler et al., 2020). This 39-week mentoring program was based on a theory of empowerment and was conducted among low-income girls in Liberia. Participation increased contraceptive use and decreased sexual activity, but did not affect pregnancy, school enrollment or completion, or experiencing sexual violence.

### Interventions Addressing Two Outcomes

#### Substance Use and SRH

Four programs (19.0%) focused on substance use and SRH. The most successful outcomes were produced by two similar trainings for youth and families—a 10-hour training based on the social development model and a 13-hour training based on risk and protective factor models (Spoth et al., 2014). Compared with the control arm, both trainings significantly increased contraception and reduced sexual activity, substance use, and substance use during sex.

The other three programs that addressed these domains produced mixed results, and there was no clear pattern in terms of length of intervention, participant characteristics, or setting that explained the variation in outcomes. For example, a culturally specific, family-based intervention based on socioecological theory consisted of nine family group sessions and 10 home visits among Hispanic parent–child pairs in the United States (Pantin et al., 2009). This program increased contraceptive use and mitigated increases over time in drug use, but did not affect sexual activity. Another program based on the theory of planned behavior and administered to youth in juvenile detention settings consisted of either a single group therapy session or a single group therapy session plus one motivational interview (Bryan et al., 2009). Both versions maintained significantly higher levels of condom use over time, but did not affect alcohol use or frequency of intercourse while drinking alcohol. A program in South Africa consisted of 18 classroom lessons taught over 2 years and was based on PYD and ecological systems theory (Smith et al., 2008). This program reduced alcohol use but did not affect condom use.

#### Substance Use and Violence

Of the four interventions that addressed substance use and violence, the most successful was a program of twelve 40-minute antidrug abuse lessons taught to 12th graders in the United States, which was based on multiple theories from behavioral therapy, social psychology, and sociology (Sussman et al., 2002). This program significantly reduced the use of alcohol, marijuana, and hard drugs, as well as weapon carrying and violent victimization.

The other three interventions addressing substance use and violence were administered to younger youth and had weaker results. For example, a 5-year program in which stakeholders in 24 communities chose evidence-based programs to implement locally with fifth-grade students significantly reduced initiation of alcohol, drugs, and violent behavior, but did not affect past-year prevalence of these behaviors (Oesterle et al., 2018). A program based on social learning and social interaction theories and consisting of group skill-building sessions and individual coaching for 100 middle school girls in foster care and their caregivers significantly reduced substance use but had no effect on delinquency, which included violent acts and damaging property (Kim & Leve, 2011). The program with the weakest results consisted of 12 lessons based on the global social influence model and was taught to middle school students in Brazil (Gusmões et al., 2018; Sanchez et al., 2017, 2018). This program reduced only one of the seven measures of drug use, reduced violent victimization but not perpetration, and did not affect alcohol use.

#### SRH and Education/Employment

Three of the four interventions addressing SRH and education/employment were implemented with girls in LMICs and were based on empowerment theories. In addition to SRH and life skills curricula, these interventions included components such as vouchers for health care services; provision of safe spaces for girls; adolescent-friendly savings accounts, activities, or incentives directed at girls’ families; and postprogram support for participants. The most successful intervention was a 6-month program in India that provided vocational skills, life skills, literacy, and SRH services (Centre for Development and Population Activities, 2001). Although specific approaches and activities varied widely in different regions, the program significantly increased contraception use, schooling, and employment. In Zambia, a 2-year program of weekly group meetings with mentors reduced transactional sex, but did not affect contraception, pregnancy, or schooling (Austrian et al., 2020). An intensive (12 hours per week for 30 months) program in Egypt increased girls’ participation in formal schooling and their literacy but did not report on behavioral SRH outcomes (Brady et al., 2007).

One intervention in the United States was conducted in schools, was based on PYD, and involved a 25-hour community service component (Allen & Philliber, 2001). This program significantly reduced pregnancy among girls and pregnancy caused by boys, and also decreased course failure and school suspensions.

#### Substance Use and Education/Employment

The only intervention that addressed this domain pair consisted of a 6-week residential treatment program based on PYD, with 3 years of mentoring follow-up (Schwartz et al., 2013). This program significantly increased earning a high school diploma or Graduate Equivalency Degree (GED), college credit, employment, and income but did not affect substance use.

## Discussion

This scoping review found 23 studies evaluating 21 theory-based programs for youth that addressed at least two different health and development domains. These programs varied considerably in their theoretical foundations, approaches, context, length, and results. While all reported significant improvement in at least one outcome or for at least one subgroup of youth, many showed mixed results either between the outcomes of interest or by subgroup. This highlights the ongoing need to determine whether there are better outcomes or a synergistic effect from targeting multiple behaviors compared with single-component interventions as well as how gender and other contextual factors may affect outcomes. While these results add to the debate between single-component and multicomponent interventions (Busch et al., 2013; Chandra-Mouli & Plesons, 2021; Haberland et al., 2018; Hale et al., 2014; Malhotra & Elnakib, 2021), important questions remain regarding implementation and outcomes.

Most programs addressed two of the health and development domains of interest, although six incorporated three domains, and one incorporated all four domains. Interventions addressing the combination of substance use and SRH were the most common, and all but one of the 12 programs that addressed violence also addressed substance use. This may suggest that certain outcomes have a greater natural affinity, due to a shared root cause, interactions between the outcomes, or one behavior having a moderating effect on another. For example, substance use is associated with increased sexual risk behaviors and negative educational outcomes (Beharie et al., 2019; Clark et al., 2020). Note that the country of implementation also was associated with program focus.

Most of the included interventions were implemented in school settings and nearly half were multiyear interventions. Most focused on the individual level, with a few also including interventions at the family, school, or community level. Very few addressed issues at the policy or structural level, which may be perceived as beyond the purview of the implementing agencies or more difficult to address. A systematic review of reviews of school-based interventions suggests that multicomponent interventions, including those addressing school policies and environment, may be more effective than interventions focused only on health education aimed at influencing individual behavior (Shackleton et al., 2016).

The most common theoretical foundations for the interventions were PYD and social learning theory. In LMICs, empowerment was the most common theory and focused on interventions for vulnerable girls. The lack of a specific theory was a common reason for exclusion from our final list, with several multibehavioral programs excluded because they were not theory based. While this may be partly due to the underreporting of the theoretical foundations of programs in journal articles (Painter et al., 2008), it may also reflect that many programs are not based on or designed with a specific behavioral change theory. Planners, evaluators, and decision-makers working on youth programs should focus greater attention on evidence-informed and theory-based approaches that respond to the priority population, setting, and desired outcomes (Brownson et al., 2009). Multilevel theories, such as the socioecological model, which address levels beyond the individual young person, should also be considered. While this assumes theory-based interventions are better, some programs that encompass a more iterative or quality improvement approach or that are developed in conjunction with youth or other community members may also have positive outcomes (Bose et al., 2021; Fakoya et al., 2021). Further research is needed to assess distinctions and outcomes of interventions using specific theories, such as the review of programs using PYD (Gavin et al., 2010). In addition, future research on specific behavioral interventions should include theory (or lack of) when reporting outcomes to build the evidence around theoretical relevance.

These results point to the need to further develop and research integrated programs, which remain less common than single-issue interventions. This may reflect several challenges, including organizational capacity, implementation issues, funding requirements and support, and other contextual or environmental factors as well as the complexity of addressing the multidimensional nature of young people’s lives and choices. Organizations may require cross-training to ensure high-quality implementation across the domains. A systematic review by Haberland et al. (2018) identified few studies that assessed implementation issues, such as dosage or program exposure and youth involvement in program design, revealing an important research gap. Other implementation issues, including fidelity and adaptations for different resource settings and populations, require further research. Similarly, research and evaluation efforts can be strengthened by developing more integrated and multidimensional measurements (Aguilera et al., 2022), assessing outcomes at different levels and time periods, and recognizing how other variables such as gender, income, and sexual orientation may interact with the intervention and the outcomes achieved.

### Strengths and Limitations

This review has some limitations. Most included studies were based in the United States, which may reflect a bias in published research. Despite searching the LILACS database, only one study was included from South America, and no Spanish-language studies meeting our inclusion criteria were found. This geographic distribution is similar to the findings from Hale’s earlier systematic review of interventions to address multiple risk behaviors (Hale et al., 2014). Other integrated programs were excluded from this review because they focused only on changes in knowledge and attitudes rather than behaviors, which may be of interest in future research and program development. Similarly, mental health issues and interventions may have considerable overlap with other health and developmental domains but were beyond the scope of this review.

Despite these limitations, this review identified a range of interventions from around the world, many of which show promising outcomes on adolescents’ health and well-being. While this review identified several successful programs, further implementation research should assess adaptations and replications of these interventions in other settings or regions of the world. Questions remain about what theories, aspects of program design, and implementation approaches are most effective for developing integrated interventions for youth. These answers can improve the efficiency of resource allocation, program quality, and replication in new settings. Further research should consider the challenges, and identify possible solutions, to implementing programs that go beyond an individual focus to a population focus. Additional integrated efforts at the policy and systems level are needed to address structural and social determinants of health, including discrimination, which affect youth health outcomes (Maness & Buhi, 2016).

### Conclusion

The results of this scoping review show that there is no “one-size-fits-all” to youth programming addressing these four health and development domains. While the identified programs had different goals and approaches, this review highlights the need for holistic interventions that teach health and technical skills and engage with multiple levels, including family and school. Developing programs based on relevant theories and building on prior efforts can help give young people the tools and support they need to feel empowered to make healthy decisions and thrive.

## Supplemental Material

sj-docx-1-heb-10.1177_10901981221130734 – Supplemental material for Integrated Theory-Based Health and Development Interventions for Young People: A Global Scoping Review Click here for additional data file. Supplemental material, sj-docx-1-heb-10.1177_10901981221130734 for Integrated Theory-Based Health and Development Interventions for Young People: A Global Scoping Review by Martha J. Decker, Abigail Gutmann-Gonzalez, Melissa Saphir, Ngoc Tram Nguyen, Qi Zhi and Claire D. Brindis in Health Education & Behavior
