# Supplementary material for: Integrated Theory-Based Health and Development Interventions for Young People: A Global Scoping Review
Source: Health Educ Behav. 2022 Oct 31;51(1):82–93. doi: 10.1177/10901981221130734 (PMC10785565; doi:10.1177/10901981221130734)
Supplement: sj-docx-1-heb-10.1177_10901981221130734 – Supplemental material for Integrated Theory-Based Health and Development Interventions for Young People: A Global Scoping Review [file sj-docx-1-heb-10.1177_10901981221130734.docx]

**PubMed Search Terms**

Each search included the filters “AND (("2000/01/01"[PDat] : "2021/07/01"[PDat]) AND (English[lang]))”.

| Search | Query | Date |
| --- | --- | --- |
| 1 | (adolescent[TIAB]) AND "health service"[TIAB]) | 15/05/15 |
| 2 | (adolescent[TIAB]) AND friendly[TIAB] | 15/05/15 |
| 3 | ("adolescent"[TIAB]) AND ("friendlIness"[TIAB]) | 15/05/15 |
| 4 | (adolescent[TIAB]) AND ("health access"[TIAB]) | 15/05/15 |
| 5 | (adolescent[TIAB]) AND ("health delivery"[TIAB]) | 15/05/15 |
| 6 | (("adolescent"[TIAB]) AND health[TIAB] AND facility[TIAB]) | 15/05/15 |
| 7 | ("adolescent"[TIAB]) AND ("health center"[TIAB]) | 15/05/15 |
| 8 | ("adolescent"[TIAB]) AND ("healthcare services"[TIAB]) | 15/05/15 |
| 9 | ("adolescent"[TIAB]) AND ("health care service"[TIAB]) | 15/05/15 |
| 10 | (("adolescent"[TIAB]) AND ("healthcare access”[TIAB]) | 15/05/15 |
| 11 | (("adolescent"[TIAB]) AND ("health care service"[TIAB]) | 15/05/15 |
| 12 | (("adolescent"[TIAB]) AND ("health care delivery"[TIAB])) | 15/05/15 |
| 13 | (("adolescent"[TIAB]) AND ("healthcare delivery"[TIAB])) | 15/05/15 |
| 13 | (("youth"[TIAB]) AND ("health service"[TIAB])) | 18/05/15 |
| 14 | (("youth"[TIAB]) AND ("friendly"[TIAB])) | 18/05/15 |
| 15 | (("youth"[TIAB]) AND ("friendliness"[TIAB])) | 18/05/15 |
| 16 | (("youth"[TIAB]) AND ("health access"[TIAB])) | 18/05/15 |
| 17 | (("youth"[TIAB]) AND ("clinics"[TIAB])) | 18/05/15 |
| 18 | (("youth"[TIAB]) AND ("health delivery"[TIAB])) | 18/05/15 |
| 19 | ("youth"[TIAB]) AND (TIAB) | 18/05/15 |
| 20 | (youth[TIAB]) AND ("health centers"[TIAB]) | 18/05/15 |
| 21 | (youth[TIAB]) AND (healthcare services[TIAB]) | 18/05/15 |
| 22 | (youth[TIAB]) AND ((health care access[TIAB]) OR (healthcare access[TIAB])) | 18/05/15 |
| 23 | (youth[TIAB]) AND ((health care delivery[TIAB]) OR (healthcare delivery[TIAB])) | 18/05/15 |
| 24 | ((teen[title/abstract]) OR (teens[title/abstract]) OR (teenager[title/abstract]) OR (teenagers[title/abstract])) AND ((health services[title/abstract]) OR (health service[title/abstract]) OR (healthcare services[title/abstract]) OR (health care service[title/abstract])) | 21/05/15 |
| 25 | ((teen[title/abstract]) OR (teens[title/abstract]) OR (teenager[title/abstract]) OR (teenagers[title/abstract])) AND ((friendly[title/abstract]) OR (friendliness[title/abstract])) | 21/05/15 |
| 26 | ((teen[title/abstract]) OR (teens[title/abstract]) OR (teenager[title/abstract]) OR (teenagers[title/abstract])) AND ((health access[title/abstract]) OR (healthcare access[title/abstract]) OR (health care access[title/abstract])) | 21/05/15 |
| 27 | ((teen[title/abstract]) OR (teens[title/abstract]) OR (teenager[title/abstract]) OR (teenagers[title/abstract])) AND (clinic[title/abstract]) | 21/05/15 |
| 28 | ((teen[title/abstract]) OR (teens[title/abstract]) OR (teenager[title/abstract]) OR (teenagers[title/abstract])) AND ((health delivery[title/abstract]) OR (healthcare delivery[title/abstract]) OR (health care delivery[title/abstract])) | 21/05/15 |
| 29 | ((teen[title/abstract]) OR (teens[title/abstract]) OR (teenager[title/abstract]) OR (teenagers[title/abstract])) AND ((health facility[title/abstract]) OR (health facilities[title/abstract])) | 21/05/15 |
| 30 | ((teen[title/abstract]) OR (teens[title/abstract]) OR (teenager[title/abstract]) OR (teenagers[title/abstract])) AND (health center[title/abstract]) | 21/05/15 |
| 31 | (young[title/abstract]) AND ((health service[title/abstract]) OR (healthcare service[title/abstract]) OR (health care service[title/abstract])) | 21/05/15 |
| 32 | (young[title/abstract]) AND (friendly[title/abstract]) | 21/05/15 |
| 33 | young[title/abstract] AND ((health access[title/abstract]) OR (healthcare access[title/abstract]) OR (health care access[title/abstract])) | 21/05/15 |
| 35 | (young[TIAB]) AND (clinics[TIAB]) | 21/05/15 |
| 36 | (young[TIAB]) AND ((health delivery[TIAB]) OR (healthcare delivery[TIAB]) OR (health care delivery[TIAB])) | 21/05/15 |
| 37 | (young[title/abstract]) AND ((health facility[title/abstract]) OR (health facilities[title/abstract])) | 21/05/15 |
| 38 | (young[TIAB]) AND (health center[TIAB]) | 21/05/15 |
